# New Insights into the Lake Chad Basin Population Structure Revealed by High-Throughput Genotyping of Mitochondrial DNA Coding SNPs

**DOI:** 10.1371/journal.pone.0018682

**Published:** 2011-04-20

**Authors:** María Cerezo, Viktor Černý, Ángel Carracedo, Antonio Salas

**Affiliations:** 1 Unidade de Xenética, Departamento de Anatomía Patolóxica e Ciencias Forenses, Instituto de Medicina Legal, Facultade de Medicina, Universidade de Santiago de Compostela, CIBERER, Galicia, Spain; 2 Archaeogenetics Laboratory, Institute of Archaeology of the Academy of Sciences of the Czech Republic, Prague, The Czech Republic; Institut Pasteur, France

## Abstract

**Background:**

Located in the Sudan belt, the Chad Basin forms a remarkable ecosystem, where several unique agricultural and pastoral techniques have been developed. Both from an archaeological and a genetic point of view, this region has been interpreted to be the center of a bidirectional corridor connecting West and East Africa, as well as a meeting point for populations coming from North Africa through the Saharan desert.

**Methodology/Principal Findings:**

Samples from twelve ethnic groups from the Chad Basin (*n* = 542) have been high-throughput genotyped for 230 coding region mitochondrial DNA (mtDNA) Single Nucleotide Polymorphisms (mtSNPs) using Matrix-Assisted Laser Desorption/Ionization Time-Of-Flight (MALDI-TOF) mass spectrometry. This set of mtSNPs allowed for much better phylogenetic resolution than previous studies of this geographic region, enabling new insights into its population history. Notable haplogroup (hg) heterogeneity has been observed in the Chad Basin mirroring the different demographic histories of these ethnic groups. As estimated using a Bayesian framework, nomadic populations showed negative growth which was not always correlated to their estimated effective population sizes. Nomads also showed lower diversity values than sedentary groups.

**Conclusions/Significance:**

Compared to sedentary population, nomads showed signals of stronger genetic drift occurring in their ancestral populations. These populations, however, retained more haplotype diversity in their hypervariable segments I (HVS-I), but not their mtSNPs, suggesting a more ancestral ethnogenesis. Whereas the nomadic population showed a higher Mediterranean influence signaled mainly by sub-lineages of M1, R0, U6, and U5, the other populations showed a more consistent sub-Saharan pattern. Although lifestyle may have an influence on diversity patterns and hg composition, analysis of molecular variance has not identified these differences. The present study indicates that analysis of mtSNPs at high resolution could be a fast and extensive approach for screening variation in population studies where labor-intensive techniques such as entire genome sequencing remain unfeasible.

## Introduction

The African Sahel together with a more southerly localized zone of savannah forms a clearly distinguishable biome. Also known as the Sudan or Macro-Sudan belt, this region displays some common linguistic features across current linguistic families [1]. The Sudan belt lacks higher mountains or other geographic barriers to migration and in genetics has been interpreted as a bidirectional corridor of human migrations [2], [3], [4]. From an ecological point of view this zone contains both high grasses in the north, and more or less dispersed shrubs and trees in the south, and comprises the natural surroundings known to humans in Africa from their early beginnings. The Sudan belt also experiences annual cycles of wet and dry seasons allowing for the coexistence of two populations with different lifestyles: nomadic pastoralists and sedentary farmers.

Approximately in the middle of the Sudan belt is the Lake Chad Basin, with Lake Chad in its imaginary centre. The Lake Chad Basin forms a remarkable ecosystem, where several unique agricultural and pastoral techniques have been developed [5]. Due to Pleistocene climatic oscillations Lake Chad has often changed in size and shape. For example, in the early Holocene, Lake Mega-Chad was formed covering a maximum surface area of 350,000 km^2^. Such a giant lake, the largest in Africa at the time, with a wealth of food resources undoubtedly attracted long-term human settlements. Lake Chad reached its current size of about 20,000 km^2^ approximately 3,000 years ago, around the time that the Sahara grew to its present size and became nearly impenetrable for humans [6]. Historically, inhabitants of the Chad Basin constantly migrated around Lake Chad in synchrony with the receding shorelines of the lake. It seems probable that in its present form Lake Chad acted as a final destination for two population movements in the Sudan belt – one from West represented by the Fulani and the other from East represented by the Arabs.

Variation in mitochondrial DNA (mtDNA) has demonstrated to be useful for the interpretation of historical and contemporary demographic events around the world, and in particular for reconstructing the evolution and origin of human populations. Most population studies carried out in Africa have been based on analysis of the control region of mtDNA, sometimes complemented with analysis of hg diagnostic coding region sites [7], [8], [9], [10], [11], [12], [13]. Although a number of complete African mtDNA genomes have been obtained and deposited in GenBank or other databases, most of these studies were focused on a phylogenetic rather than a demographic perspective [14], [15], [16].

On the other hand, variation in mtDNA is commonly analyzed using standard sequencing procedures targeting the first and/or the second hypervariable regions (HVS-I/II) or the whole control region (including HVS-I/II). Analysis of coding region mtDNA SNPs using minisequencing is another common approach [17], [18], [19], [20], [21]. However, this technique is inadequate for genotyping large amounts of SNPs. Recently, Cerezo et al. [22] reported a novel MassARRAY SNP genotyping system for genotyping the large number of SNPs located in the coding region of mtDNA using MALDI-TOF. In this work, we present the first population application of this technique, genotyping 542 samples from 12 different ethnic groups of the Lake Chad Basin.

## Materials and Methods

### Ethics Statement

Oral informed consent was required for the samples, and all of them were anonymized. The study was approved by the Ethical committee of the University of Santiago de Compostela. The study also conforms to the Spanish Law for Biomedical Research (Law 14/2007- 3 of July).

### Population samples

We analyzed 542 individuals from 12 different ethnic populations sampled around the Lake Chad Basin. Most of these samples (*n* = 441; 80%) were previously reported for the HVS-I segment and selected Restriction Fragment Length Polymorphisms (RPLPs) in Černý et al. [3]. Information about the ethnic adscription and the rationale for sampling collection is provided in [3] (see also Table 1). Briefly, we have analyzed the following population samples: Hide (*n* = 47), Kotoko (*n* = 62), Mafa (*n* = 57), Masa (*n* = 41), Buduma (*n* = 30), Chad Arabs (*n* = 27), Shuwa Arabs (*n* = 39), Fali (*n* = 40), Bongor Fulani (*n* = 50), Tcheboua Fulani (*n* = 40), Kanembu (*n* = 50) and Kanuri (*n* = 59). DNA extract were then submitted to the laboratory of Santiago de Compostela where the genotyping was carried out.'

**Table 1:** Populations analyzed in the present study.

Population	Code	*n*	Geographical Region	Language branch/Language Family	Lifestyle
Hide	Hi	47	Northern Cameroon	Chadic/AA	Sedentary
Kotoko	Ko	62	Northern Cameroon	Chadic/AA	Sedentary
Mafa	Mf	57	Northern Cameroon	Chadic/AA	Sedentary
Masa	Ms	41	Northern Cameroon	Chadic/AA	Sedentary
Buduma	Bu	30	South-eastern Niger	Chadic/AA	Sedentary
Chad Arabs	CA	27	Southwestern Chad	Semitic/AA	Nomadic
Shuwa Arabs	SA	39	Southwestern Chad	Semitic/AA	Semi-nomadic
Fali	Fa	40	Northern Cameroon	Adamawa-Ubangui/NC	Sedentary
Bongor Fulani	BF	50	Southwest Chad	Atlantic/NC	Nomadic
Tcheboua Fulani	TF	40	Northern Cameroon	Atlantic/NC	Nomadic
Kanembu	Kb	50	South- western Chad	Saharan/NS	Sedentary
Kanuri	Ka	59	North-eastern Nigeria	Saharan/NS	Sedentary

NOTE: AA  =  Afro-Asiatic; NC  =  Niger-Congo; NS  =  Nilo-Saharan.

Most of this information is available in Table 1 of Černý et al. [3] and it is replicated here for the sake of clarity.

DNA extraction of new samples was carried out as described in Černý et al. [3]. All samples analyzed in the present study were previously subjected to whole genome amplification (WGA) using the Genomiphi v.2 kit (GE Healthcare Life Sciences; Uppsala, Sweden) according to the manufacturer's protocol. Only 1 µl of the original extracted DNA (at least >10 ng/µl) was used for WGA. The WGA product was subsequently diluted 1∶16 in water and then directly used for MALDI-TOF MS genotyping.

### MALDI-TOF MS mtSNP genotyping

All samples were genotyped for a set of 230 mtSNPs using the technology described in Cerezo et al. [22]. Sixty of the samples (11%) were already genotyped for the whole set of mtSNPs [22] (Table 2).

**Table 2:** mtDNA diversity in the Chad Basin.

		HVS-I						mtSNP						HVS-I plus mtSNP					
	*n*	*k*	*k/n*	*S*	*h*	*Π*	*M*	*k*	*k/n*	*S*	*h*	*π*	*M*	*k*	*k/n*	*S*	*h*	*π*	*M*
**Ethnic group**																			
Hide	47	38	0.81	51	0.991±0.006	0.025±0.002	8.60	25	0.53	70	0.963±0.013	0.051±0.003	11.61	38	0.81	121	0.991±0.006	0.036±0.002	20.21
Kotoko	62	33	0.53	51	0.961±0.014	0.020±0.002	6.91	29	0.47	72	0.947±0.016	0.051±0.003	11.61	40	0.65	123	0.977±0.010	0.033±0.002	18.52
Mafa	57	37	0.65	62	0.980±0.008	0.023±0.002	7.80	32	0.56	71	0.961±0.012	0.047±0.012	10.52	42	0.74	133	0.985±0.007	0.032±0.002	18.32
Masa	41	35	0.85	43	0.991±0.008	0.022±0.002	7.34	26	0.63	67	0.971±0.012	0.049±0.003	11.01	36	0.88	110	0.993±0.008	0.032±0.002	18.35
Buduma	30	22	0.73	43	0.968±0.021	0.023±0.002	7.74	18	0.60	57	0.954±0.021	0.041±0.003	9.34	22	0.73	100	0.968±0.021	0.030±0.002	17.09
Chad Arabs	27	20	0.74	36	0.963±0.023	0.020±0.002	6.78	19	0.70	54	0.969±0.018	0.046±0.003	10.52	22	0.81	90	0.980±0.017	0.030±0.001	17.30
Shuwa Arabs	39	29	0.74	44	0.980±0.011	0.018±0.001	6.04	23	0.59	54	0.968±0.012	0.042±0.002	9.57	31	0.79	98	0.987±0.009	0.027±0.001	15.61
Fali	40	23	0.58	44	0.962±0.014	0.022±0.002	7.43	22	0.55	55	0.953±0.017	0.050±0003	11.32	26	0.65	99	0.971±0.013	0.033±0.002	18.75
Bongor Fulani	50	27	0.54	35	0.937±0.023	0.020±0.001	6.87	24	0.48	50	0.937±0.023	0.045±0.002	10.19	32	0.64	85	0.954±0.021	0.030±0.001	17.05
Tcheboua Fulani	40	21	0.53	42	0.953±0.016	0.021±0.002	7.31	19	1.30	52	0.942±0.017	0.043±0.002	9.87	27	0.68	94	0.971±0.014	0.030±0.002	17.19
Kanembu	50	38	0.76	57	0.989±0.006	0.026±0.002	8.80	31	0.62	68	0.978±0.008	0.049±0.003	10.99	42	0.84	125	0.993±0.006	0.035±0.002	19.79
Kanuri	59	47	0.80	55	0.990±0.006	0.022±±0.002	7.44	35	0.59	82	0.976±0.008	0.047±0.003	10.74	51	0.86	137	0.994±0.005	0.032±0.002	18.18
TOTAL	542	248	0.46	117	0.991±0.001	0.022±0.001	7.44	143	0.26	136	0.977±0.002	0.049±0.001	11.08	315	0.58	253	0.995±0.001	0.033±0.000	18.53
**Lifestyle**																			
Sedentary	386	195	0.51	107	0.990±0.001	0.022±0.001	7.57	112	0.29	128	0.975±0.002	0.050±0.001	11.22	237	0.61	235	0.994±0.001	0.033±0.001	18.79
Nomadic	156	83	0.53	73	0.979±0.005	0.021±0.001	7.01	59	0.38	91	0.969±0.0006	0.046±0.001	10.45	101	0.65	164	0.987±0.004	0.031±0.001	17.46
**Language Family**																			
Niger-Congo	130	60	0.46	61	0.964±0.007	0.021±0.001	7.28	45	0.35	80	0.956±0.009	0.047±0.001	10.56	75	0.58	141	0.980±0.006	0.031±0.001	17.85
Nilo-Saharan	109	80	0.73	74	0.993±0.002	0.024±0.001	8.25	55	0.50	96	0.978±0.005	0.050±0.002	11.25	88	0.81	170	0.995±0.002	0.034±0.001	19.49
Afro-Asiatic	303	151	0.50	105	0.990±0.001	0.021±0.001	7.23	101	0.33	119	0.977±0.003	0.049±0.001	10.98	192	0.63	217	0.995±0.001	0.032±0.001	18.21
**Geographical region**																			
North	267	143	0.54	98	0.990±0.002	0.021±0.001	7.26	96	0.36	117	0.978±0.003	0.049±0.001	11.00	1789	6.70	215	0.994±0.001	0.032±0.001	18.26
South	275	143	0.52	87	0.987±0.002	0.023±0.001	7.76	83	0.30	112	0.970±0.004	0.049±0.001	10.96	170	0.62	199	0.993±0.001	0.033±0.001	18.72

NOTE: *n*  =  sample size; *k*  =  number of different sequences; *S*  =  number of segregating sites; *h*  =  haplotype diversity; *π*  =  nucleotide diversity; *M*  =  average number of pairwise differences (mismatch observed mean). For all of the samples, the common segment of the HVS-I region analyzed ranges from position 16030 to 16370 (with the exception of samples #Fa108 and #Hi14 that present sequence ranges outside 16030–16370 and were therefore eliminated from the analysis; see Table S2).

**Figure 1:**
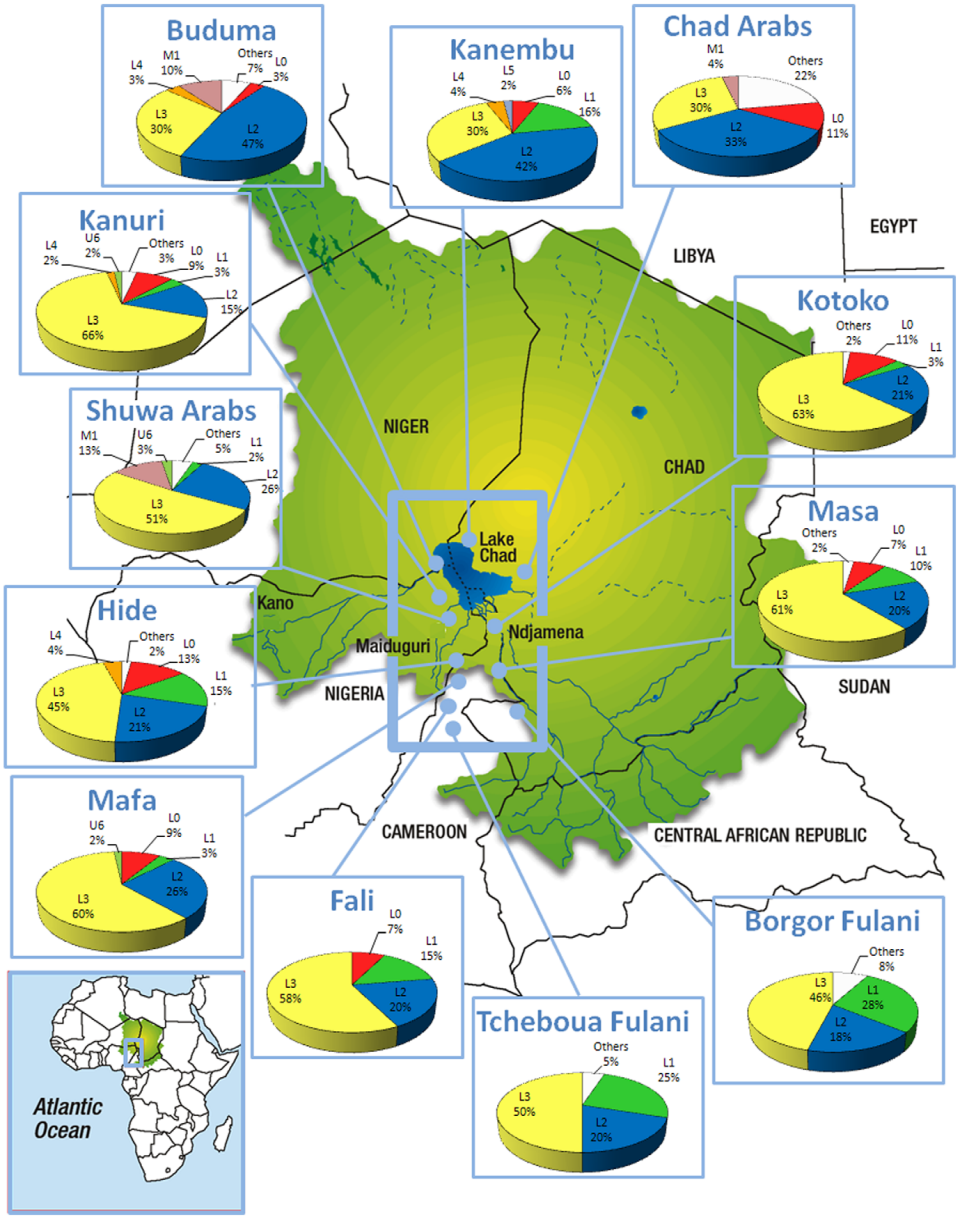
Map of the Lake Chad Basin showing frequencies of the main African hgs in the different ethnic groups analyzed.

The two phylogenetic trees in Figure 1 and 2 of Cerezo et al. [22] indicate all of the mtSNPs genotyped in the present study, including diagnostic control region variants.

**Figure 2:**
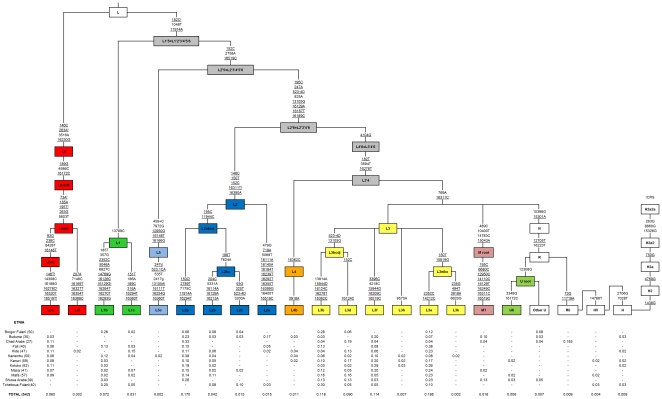
Phylogeny of African hgs at a medium level of phylogenetic resolution and (below branches) counts of these hgs for the different ethnic groups. Bottom of the figure: population labels have in brackets the sample sizes; numbers below branches indicate the hg relative frequencies in each population group and in the total sample size (row “Total”); therefore, each row sums to 1. The counts for the maximum level of resolution are provided in Table S2; the full phylogenetic tree for the SNPs considered in the present study is provided in Cerezo et al. [22]. All positions in the tree refer to the revised Cambridge Reference Sequence (rCRS; [49]); all positions are transitions unless a letter indicates a transversion. Underlined positions are parallel mutations within this tree, while “!” indicates a back mutation. A deletion is indicated as “del”, while “+” indicates an insertion.

Assessment of the genotyping quality was carried out by replicating some samples from the Chad Basin in different runs plus six good quality DNA samples from the CEPH (Centre d'Etude du Polymorphisme Humain; http://www.cephb.fr/en/cephdb/), namely, NA10830, NA10831, NA10860, NA10861, NA11984, NA12147 (that were used as positive controls). Table S1 summarizes information on call rates per mtSNP. We have not detected genotyping inconsistencies among 7,436 replicated genotypes. For 77% of the mtSNPs the calling rate was above 90%; which can be considered quite acceptable if we take into account that all the samples were collected several years ago (some of the buccal swabs are now more than 10 years old). Some mtSNPs however virtually failed (e.g. 5128G; 5746A) or yielded poor results (650C; 9545G). More information is available in Table S1.

Given that the majority of the failed mtSNPs did not occur at the final branches, there were no problems to assign lineages to their maximum known level of phylogenetic resolution.

### Standard sequencing analysis

All phylogenetic inconsistencies observed using MALDI-TOF MS were automatically sequenced using the protocol described in Álvarez-Iglesias et al. [20] and as indicated in Cerezo et al. [22]. New samples were also sequenced for the control region (see Table S2).

### Nomenclature

African phylogeny and nomenclature is very complex and has been elaborated during the last decade based on the control region and complete genome sequencing efforts [8], [9], [13], [14], [15], [23]. All of these phylogenetic efforts have been compiled in the Phylotree project (http://www.phylotree.org/; mtDNA tree Build 11; 7 Feb 2011) [24].

### Statistical analysis

DnaSP v.5 [25] was used to compute diversity indices, including nucleotide and haplotype diversity and the average number of nucleotide differences. Arlequin 3.5.1.2 [26] was used to conduct analysis of molecular variance (AMOVA); the significance of the covariance components associated with different levels of genetic structure was tested using a non-parametric permutation procedure [26].

Principal Component Analysis was carried out on population hg frequencies and using R (http://www.r-project.org/).

Lamarc (Likelihood Analysis with Metropolis Algorithm using Random Coalescence) [27] was used to estimate: (i) θ, which for females it is expected to be equal to 2*N*_e_µ for neutral mutations in mtDNA (where *µ* is the neutral mutation rate per generation and *N*_e_ the effective population size of females), (ii) population growth as *g* = -(lnθ_t_/lnθ_present day_)/*t*, where *g* is expressed as the relationship between θ at a time *t*>0 in the past and θ at the present (*t* = 0), and (iii) migration rate, defined as *M* = *m*/µ, where *m* is the immigration rate per generation), between the 12 ethnic groups used in the present study and using information from mtSNPs. Estimates were obtained for three independent replicates using a Bayesian framework. The jModelTest v.0.1.1. [28] software (with default heating and burn-in parameters) was used to obtain the base frequencies, mutation parameters and the best mutation model (according to the Akaike information criteria), which (for our data) was the general time reversible (*GTR*) model. Estimates from iModelTest were obtained using three replicates using 10 initial chains (sampling interval of 20 and burn-in period of 1000) and two final chains (sampling interval of 20 and burn-in period of 1000). Transition:transversion rate was set to 30.

Some caveats should be considered in regards to the demographic estimates obtained. The mtDNA is in reality a single locus and therefore all the values should be taken with caution: “*For estimation of Theta and migration rate it is possible to get results with one region but they will improve markedly with more; doubling the number of regions nearly doubles the available information. Estimation of growth rate is very poor with less than 3 unlinked regions and particularly benefits from having more.*” (http://src.gnu-darwin.org/ports/biology/lamarc/work/lamarc-2.1.2b/doc/html/data_required.html). On the other hand, sample sizes for some of the groups are relatively low and therefore the impact on the different estimates is unpredictable. Last but not least, the neighboring source populations for the Chad Basin are not represented in the mathematical model (e.g. East, North, western Africa, etc); therefore, we are committed to assume a simplistic model were the provenance of the different lineages comes from one of the 12 ethnic groups considered.

For all of these computations and in order to account for missing data, failed SNPs were imputed according to known phylogeny. Given the fact that there exists a robust mtDNA worldwide phylogeny based on entire mtDNA genomes (>8,700), the phylogenetic-based approach for imputation seems more reliable than those based on e.g. metrics for linkage disequilibrium [29].

## Results

### Genetic diversity in the Chad Basin populations

Several diversity indices have been computed for the 12 ethnic groups analyzed in this study. These indices have been obtained both individually for HVS-I and mtSNPs, and in combination for HVS-I plus mtSNPs (Table 2). With few exceptions, haplotype diversity yielded slightly higher values for HVS-I than for mtSNPs whereas nucleotide diversity was approximately twice as large for the mtSNPs as for HVS-I. Diversity values are very heterogeneous among the 12 population samples analyzed. The Hide sample shows high values of diversity independently of the mtDNA segment analyzed, whereas the opposite pattern was observed in the two Fulani samples. In general, the four nomadic populations included in this study have lower diversity values than the sedentary populations (Table 2).

Some minor differences may be related to language family, providing an explanation for why the Niger-Congo family has lower diversity values than the other two groups (Table 2), but the latter probably reflects the presence of the low diversity characterizing the two nomadic Fulani groups. No differences were observed in diversity values between populations located in the north (the Shuwa and Chad Arabs, Kanuri, Buduma, Kanembu, and Kotoko) *versus* those located in the south (the Bongor and Tcheboua Fulani, Hide, Mafa, and Masa; see map in Figure 1).

The diversity values obtained for the combined HVS-I plus mtSNPs are approximately an average of the values obtained for the two segments individually for nucleotide diversity and the average number of nucleotide differences, but, as expected, are slightly higher for haplotype diversity in most of the groups.

### Phylogeography of the populations in the Chad Basin

The graphs in Figure 1 show the distribution of main African hgs in the Chad Basin. This broad hg classification clearly indicates substantial heterogeneity in the region. For instance, the Kotoko, Masa, Kanuri, and Mafa have frequencies of L3 haplotypes above 60%, in contrast with frequencies of only 30% for the Kanembu, Buduma and Chad Arabs. The Shuwa Arabs and both Fulani populations (Tcheboua and Bongor) do not have L0 haplotypes, which are approximately 11% in the Kotoko and 13% in the Hide. L2 is above 40% in the Buduma and Kanembu, but only 20% or less in the Masa, Kanuri, Fali, and both of the Fulani populations. Haplogroup M1 is present only in both Arab populations and the Buduma. Percentages of non-Sub-Saharan lineages vary also among ethnic groups (included in the category “Others” in Figure 1).

Figure 2 shows a medium resolution phylogeny indicating hg frequencies in the 12 groups genotyped. The most common sub-lineages in all the Chad Basin populations are L2a, L3b, L3f, and L3e.

Haplogroup frequencies measured to the maximum obtainable resolution for the mtDNAs genotyped in this study are shown in Table S3. The data indicate that the sub-hgs, L3b1a and L3e5, are the most common lineages in the Chad Basin (both accounting for >17% of the total sample). These two lineages are present in nearly all of the populations included in this study with the exception of the Chad Arabs (both L3b1a and L3e5) and the Buduma (L3e5). The unusual high frequency of L3e5 in the Chad Basin could be explained by a local expansion; however, because nearly all of the population samples analyzed carry L3e5 mtDNAs, it is more likely that this event occurred before the ethnogenesis of the region. At this level of resolution, it is remarkable that some sub-lineages are frequent in some populations but nearly absent in the rest. For instance, L1b1a appears in the two Fulani groups with frequencies greater than 18%, but with significantly lower frequencies in the other groups (below 6% in the Kanuri, and less than 3% in the other populations). L3b1b is also observed with high frequency in the Fulani Tcheboua (15%), but only appears with low frequency in the Fulani Bongor (4%) and is absent in the rest of the populations. Chad Arabs account for all the R0a mtDNAs in the Chad Basin (19%), in agreement with the high frequency of R0a reported in the Arabian Peninsula [30] and especially in its Southern tip and Socotra island [31]. The whole genome of two of these samples was recently reported [32] and classified within the widespread subclade R0a2f characterized by a substitution at position 8251. No mtDNAs of Eurasian ancestry have been observed in samples from the Fali, Kanembu, Mafa, and Masa. The typical North African lineages (M1 and U6; 2% in the total sample) are mainly observed in the two Arab groups. Most of the M1 lineages likely come from the Mediterranean instead of East Africa. For instance, four Shuwa Arabs belong to M1a1 and another within the sub-branch M1a1a (as indicated by a transition at position 14182 and a reversion at position 16249); the distribution of M1a1 is mainly Mediterranean. The Chad Arab sample #AC92 and the two Buduma #Bu87 and #Bu89 belong to the M1a3 branch, which has a predominant Mediterranean distribution. Finally, two other Buduma samples belong to M1a3, also mainly of a Mediterranean distribution [23]. The three mtDNAs belonging to U6 were found in one Shuwa Arab, one Kanuri and one Mafa, with the one in the Kanuri belonging to U6a5, again a Mediterranean branch. More interestingly, the U6b lineage found in the Mafa is of the so called “Canarian Branch”, indicating that perhaps the Chad Basin could participate in the demographic wave that originally moved U6 hg towards the Canary Island from East Africa.

### Analysis of molecular variance in the Chad Basin

Analyses of molecular variance (AMOVA) were carried out on the 12 Chad Basin populations analyzed in this study using the following grouping schemes: all of the populations individually, populations grouped by language family, and populations grouped according to their locations in the North or the South of the Chad Basin (Table 3). Most of the genetic variation (∼96%) was found to occur within populations, whereas variation between populations accounted for only 4%. These values were virtually the same independently of the grouping scheme. Genetic variation among these groups is therefore below the inter-population differentiation reported to exist on the African continent (∼12%; see [3]). The level of molecular resolution does not seem to be an influencing factor in the apportioning of genetic variance in the Chad Basin (Table 3), although mtSNPs do seem to contribute a subtle increment to the genetic variation.

**Table 3:** Apportioning of genetic variance considering different genomic regions (HVS-I, mtSNPs, and both in combination) and groups (populations, language families and geography).

	HVS-I		mtSNPs		HVS-I+ mtSNPs	
	Among populations*1	Within populations*1	Among populations*1	Within populations*1	Among populations*1	Within populations*1
All populations	3.24	96.76	3.83	96.17	3.59	96.41
Language	3.82*2	96.18	4.28*2	95.71	4.10*2	95.90
Geography	3.50*2	96.50	4.03*2	95.97	4.39*2	95.61

*1 *P-*values are below 0.0000 using a significance test based on 20.000 permutations.

*2 Among groups + Among populations within groups.

### Principal Component Analysis of the Chad Basin populations

**Figure 3:**
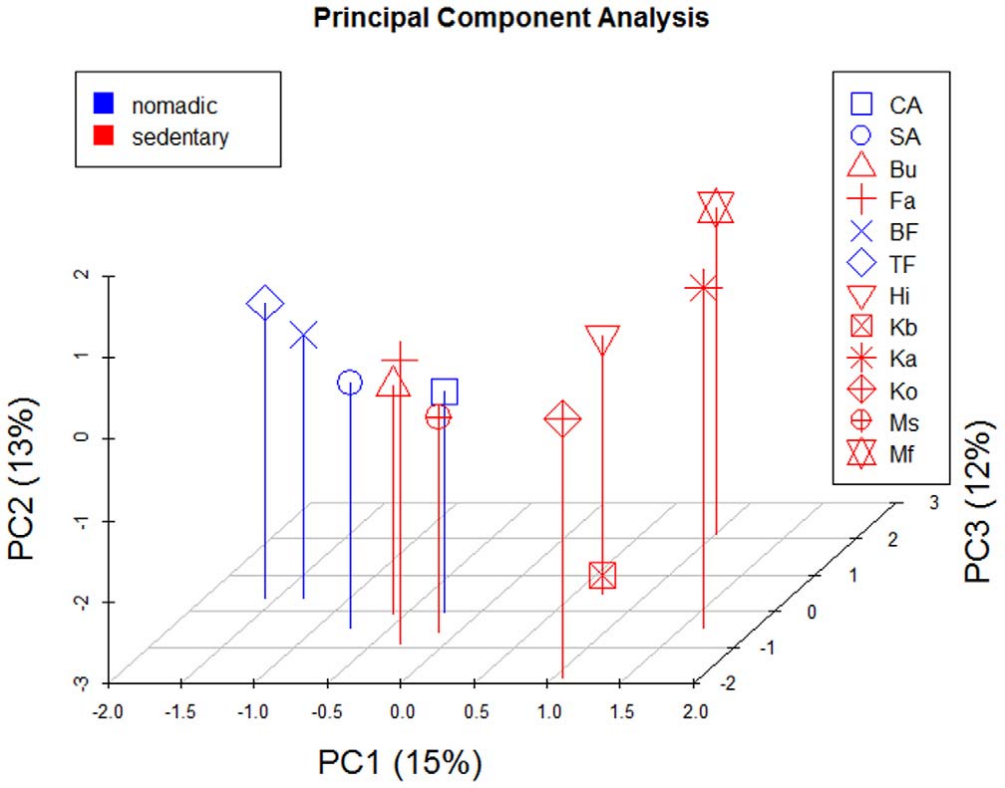
PC plot of ethnic relationships based on hg frequencies. Percentage values in brackets refer to the amount of variation accounted by the first three principal components (PC1, PC2, and PC3). Codes for populations are as indicated in Table 1. Nomadic populations are plotted in blue while sedentary ones are plotted in red.

PCA was carried out based on hg frequencies to the maximum level of resolution. The three first components account for a total of ∼40% of the variation, and it shows notable divergence between the different ethnic groups from the Chad Basin. Thus, the first principal component (PC1), which accounts for 15% of the variation, locates Mafa and Kanuri in one side of the plot, and the two Fulani populations in the opposite pole. PC2 (13%) shows also the Mafa in one side of the plot and the Kanembu in the other extreme. PC3 (12%) displays again Mafa in one pole opposed to the Kotoko. There are not unique features in the Mafa that makes this population different to the other ethnic groups, but an accumulative effect of several differences in hg frequencies (some of them are more pronounced than others e.g. high frequency of hgs L2b2, L3d1d, L3e2). Apart from the two Fulani samples, the two Arab ones are proximal in the plot indicating a close maternal phylogenetic relationship. The most distinctive features of the two Arab populations compared to the other Chad populations are the presence of non sub-Saharan lineages, such as R0a or M1 hgs.In agreement with the analysis of the different genetic diversity metrics, it is interesting to note that the nomadic populations are more tightly grouped in the scatter plot than the sedentary ones (Figure 3), mirroring their more reduced genetic diversity.

### Population growth, effective population size and migration rates

We further estimated the population mutation parameter, θ, which in conjunction with the average mtDNA mutation rate was used to infer the effective population sizes of the different Chad Basin populations assuming a neutral model of molecular evolution (Table 4). For an average entire genome mutation rate of 1.655 × 10^−8^ base substitution per nucleotide per year [33], female effective population size ranges from 359,200 in Masa to 5,423,000 in Buduma (Table 4). Curiously, three out of four populations (Chad Arabs, and Bongor and Tcheboua Fulani) showed negative growth rates, while others have positive values, such as the Buduma and the nomadic Shuwa Arabs (Table 4).

**Table 4:** Inter-population migration rates, population growth, and effective population sizes for the different ethnic groups from the Chad Basin.

	CA	SA	Bu	Fa	BF	TF	Hi	Kb	Ka	Ko	Ms	Mf
**Migration rates**												
CA	−	163.3	110.4	173.3	216.2	119.3*	67.0	166.9*	70.5	112.1	96.1	76.1
SA	88.5	−	41.9	152.0	231.9	83.6	69.1*	28.2	107.1	93.2	109.4	74.7
Bu	135.8	36.1	−	108.8	79.5	66.1	94.1	346.1*	53.1	104.0*	89.2	159.9*
Fa	59.9*	121.2	43.6	−	163.9	122.7	243.9	123.1	87.1	87.1	59.0	86.0
BF	189.7*	270.7	23.7	319.2	−	140.3	19.9	45.3	69.8	51.4	189.9	63.0
TF	60.2	61.9	36.6	162.8	154.0	−	117.9	50.3	95.4	76.7	153.0	50.6
Hi	74.1	143.5*	139.3	55.9	24.9	201.6	−	163.7*	71.0	198.5*	486.9*	152.6*
Kb	404.9*	81.0	575.2*	232.4	25.6	69.4	96.9*	−	109.1	76.7	82.6	112.7*
Ka	69.0	161.4	99.3	156.5	361.1	119.1	66.6	144.1	−	138.5	647.0	63.6
Ko	98.1	97.0	73.9	110.1	53.7	95.0	116.2*	113.7	100.1	−	202.9*	113.1
Ms	140.5	219.9*	91.5	327.5	91.0	174.9	62.9*	74.8	104.0	195.3	−	146.4
Mf	146.4	105.8	136.0	79.5	130.1	115.8	193.8*	144.2	27.5	103.1	90.2	−
**Theta**	0.017*	0.026*	0.181*	0.014*	0.012*	0.018*	0.025*	0.038*	0.066*	0.023*	0.012*	0.050*
***N*** **_e_** (females)	520,600	769,100	5,423,000	414,700	362,800	545,800	740,400	1,136,000	1,977,900	677,100	359,200	1,498,000
**Growth**	−138.66	252.15	311.13*	150.23*	−48.97*	−64.94	91.96*	41.77	64.25	20.38	131.07	147.53*

Migration rates: numbers indicate the gene flow from each population group (as indicated in the first column) into the other populations (as indicated in the first row); for instance, 88.5 would be the migration rate from Shuwa Arabs into Chad Arabs. Population codes are as indicated in Table 1. Stars indicate estimates that have to be taken with care due to limited sample sizes (as inferred by Lamarc).

There are not unique features that would explain the observed migration rates values (although not all should be considered as reliable estimates; see M&M; and Table 4). Thus, for instance, the highest migration rate was obtained for Kanuri into Masa (Table 4); from a phylogeographic point of view these two populations share the highest amount of sub-clades (Table S3), and they are displayed together for the PC3 (and to a minor extent for the PC2) in the PCA. Bongor Fulani have the highest frequencies for hg L1b1a and L3b1a, which could explain their influence in the Fali.

## Discussion

The values of the diversity indices computed for the HVS-I and the mtSNPs show clear-cut differences, mirroring the fact that haplotype diversity is enriched in the HVS-I segment by the presence of rare (or private) variants, whereas the agglomeration of identical sequences into different hgs (possibly suffering from bias in mtSNPs selection) enriches the nucleotide diversity. Therefore, values computed using these different mtDNA segments summarize different aspects of the molecular diversity in populations. In fact, diversity values of HVS-I and mtSNPs for the 12 different ethnic groups analyzed moderately correlate for the haplotype diversity (*h*, r^2^ = 0.90), but very poorly correlate for the nucleotide diversity (*π*, r^2^ = 0.42). Thus, one can speculate different demographic scenarios for each population according to their differential diversity values. For instance, nomadic or semi-nomadic populations tend to experience loss of diversity by genetic drift (assuming moderate admixture with those populations they meet sporadically), reducing both haplotype and nucleotide diversities. Provided that the gene flow is negligible for a given effective population size, the HVS-I region of more ancestral nomadic populations would be expected to retain more haplotype diversity (due to the presence of more rare variants) than younger nomadic groups. Thus, although the (semi-)nomadic populations (the Chad Arabs, Shuwa Arabs, Bongor Fulani, and Tcheboua Fulani) all have very low nucleotide and haplotype diversity values compared with sedentary populations, the two Arab populations retained higher values of haplotype diversity in the HVS-I segment than the Fulani (this signal is not as clear for nucleotide diversity in the HVS-I segment, possibly due to the low inter-population differences observed for all of the population groups analyzed). This hypothesis is compatible with the fact that nomadic populations as a whole have lower diversity than sedentary groups (Table 2). Finally, values for the average number of pairwise differences and nucleotide diversity are highly correlated, as expected given that both indices are based on the same principles.

Demographic inferences carried out only using summarizing indices (such as nucleotide and haplotype diversities) have to be considered with caution because in reality each human population defined only by ethno-linguistic criteria is composed of an amalgamation of genetic lineages of different ages and origins, and therefore, none has a simple past.

Haplogroup patterns vary substantially among the different ethnic groups studied. In some, hg composition seems to correlate well with historical documentation and their known demographic past. Thus, the presence of R0a only in Chad Arabs is expected given the high frequency of this hg in Southern Arabia [32]. In addition, the M1 sub-lineages observed in our samples have a mainly Mediterranean distribution, and are exclusively found in the two Arab populations and the Buduma (also located in the northern Chad Basin). The spread of this hg to the African Sahel (and possibly further into the Chad Basin) might have been mediated by the Tuareg nomads [34]. Also, the presence of L1c sub-lineages in the Hide (Cameroon; Chad Basin) compared with the rest of the populations indicate narrow contact of this population with Central African populations (including Pygmy populations), where this lineage is found with high frequency [7], [8], [16].

The few Eurasian profiles observed in the Chad Basin did not cluster in any particular ethnic group. Their control region segments are not informative from a phylogeographic point of view, and these sequences are broadly distributed around Eurasia. The only exception is the U5b1 HVS-I profile T16189C C16192T C16270T C16320T that is detected mainly in Africa [35] and is a perfect match with a sample from Spain (https://www.policia.es/cgpc/index.htm).

With the exception of a few population studies based on complete genomes [36], [37] or coding region segments [38], [39], [40], most of the genotyping studies carried out to date were based on control region sequences [8] and/or mtSNPs at a low to moderate level of hg definition [18], [19], [20], [21], [41], [42]. Some other studies [15], [43], [44], [45] focused on phylogenetic issues by genotyping selected branches of the mtDNA tree, but did not consider the population as a whole. The mtSNPs genotyped in this study were designed to identify mtDNAs of African ancestry to the maximum level of molecular resolution provided by known phylogeny based on complete genome sequences. In theory, the 230 mtSNPs should be able to discriminate among 147 different terminal branches of the Sub-Saharan phylogeny (L-hgs), along with dozens of intermediate hgs, and other African non-L branches (such as sub-hgs of U6 or M1). Moreover, the mtSNPs allow for a more rigorous classification of mtDNAs into hgs due to the (average) low mutation rate characterizing these SNPs compared with the mutation rate in the control region [33].

Analyses of mtSNPs in combination with sequencing information (control region) has provided new insights regarding population features of the Chad Basin populations [3], and open new perspectives for new pan-African phylogenetic studies as well as for the reconstruction of the patterns of Trans-Atlantic slave trade into America:

- Since the mtSNPs used in the present study were designed to detect major and minor branches of the phylogeny, while the control region variation analyzed previously [3] accounts for both (unbiased) common and rare variants, the evolutionary histories told by both sets of markers are different. Thus, as dissected in the present study, different styles of life (nomadic *versus* sedentary populations) can leave different signatures in the two sets of markers.
- We have estimated for the first time different demographic parameters of the Chad Basin populations, including *N*_e_, population growth, and migration rates. Nomadic populations show signals of negative growth (as also indirectly indicated by diversity metrics), which not always coincide with those that have higher *N*_e_; in fact, both parameters are just moderately correlated (r^2^ = 0.59). However, larger sample sizes would be required in order to yield solid figures for all these parameters.
- Analysis of mtSNPs have allowed to reveal new phylogeographic features in the Chad populations, not discussed previously [3]. Given that this is the first study analyzing a pan-African mtSNP hg panel to a population level (with the only exception of a test sample analyzed previously [22]), it is still not possible to make inferences concerning the gene flow of neighboring source populations to the different Chad ethnic groups; however, the present study provides a high resolution hg map for future African studies.
- It has been previously demonstrated [7], [9], [46] that only broad patterns of variability can be established in Africa with the current mtDNA data (basically HVS-I segments); therefore, tracking ‘African-American’ lineages to particular African regions might be fraught with problems due to the low level of genetic resolution. Analysis of mtSNPs, as undertaken in the Chad Basin, could help to achieve new insights into the patterns of Atlantic slave trade.
- Analyzing mtSNPs to a high level of hg resolution in populations allows a better selection of mtDNAs for further entire genome sequencing or for the design of multiplex mtSNP panels of interest in population, medical, and forensic genetics [20], [21], [47], [48].

In conclusion, given that genotyping mtSNPs is straightforward compared with the intense effort demanded by sequencing complete genomes, the present study opens the door to more ambitious pan-African studies that would improve our knowledge on the mtDNA phylogeography in this continent.

## Supporting Information

Table S1 **mtSNP calling rates and number of phylogenetic inconcistencies in the global dataset. Mutation hits in Soares et al** [**33**] **are also given for the phylogenetic inconsistencies.**
(XLS)

Table S2 **mtSNP genotypes and control region data for the 542 individuals from the Chad Basin region genotyped.**
(XLS)

Table S3 **Haplogroup frequencies in all the population samples analyzed at the maximum level of resolution provided by the control region and the mtSNPs.**
(XLS)
